# Methodological Insights into Implementing cellular automata models for simulating seagrass dynamics: Responses to global change effects

**DOI:** 10.1016/j.mex.2024.102936

**Published:** 2024-08-28

**Authors:** Pedro Beca-Carretero, Marlene Meister, Mirta Teichberg, Agustin Moreira-Saporiti, Fabian Schneekloth, Hauke Reuter

**Affiliations:** a Leibniz Centre for Tropical Marine Research, Bremen, Germany; b Centro de Investigación Marina, Facultad de Ciencias del Mar, Universidad de Vigo, 36310 Vigo, Spain; c Ocean Acoustics Group, Alfred Wegener Institute Helmholtz Centre for Polar and Marine Research, Bremerhaven, Germany; d The Ecosystems Center, Marine Biological Laboratory (MBL), Woods Hole, MA, USA; e Faculty for Biology and Chemistry, University of Bremen, Bremen, Germany

**Keywords:** Mechanistic models, Ruled-based models, Climate change, Invasive species, Nutrient regimes, Modeling, Plants, Marine ecosystems, Mediterranean Sea, East Africa, Tropical species, Cellular automata models

## Abstract

This study introduces an innovative methodology employing Cellular Automata (CA) models to simulate seagrass dynamics in response to global environmental changes. The primary objective is to outline a procedural framework for constructing and deploying CA models applied to seagrass ecosystems, and potentially to other marine or terrestrial environments. The methodology encompasses various components, including conceptualization, workflow delineation, model parameterization, and execution steps. By utilizing Mediterranean and Zanzibari (East Africa) seagrass ecosystems as case studies, we demonstrate the versatility and applicability of the proposed approach across diverse geographical regions, species composition and model components. Through these case studies, we demonstrated how CA models can effectively capture the dynamics of seagrass communities subjected to climate change, invasive species, and nutrient regimes. Despite its strengths, the proposed CA model has limitations, including parameterization complexity and uncertainties related to species-specific environmental thresholds, growth rates and species interactions, alongside the difficulty of validating our models with real-world scenarios. Addressing these limitations in future studies will enhance the model's accuracy and applicability. This study serves as a foundation for future research in other regions and ecosystems, facilitating a better understanding of the complex interactions driving ecosystem dynamics*.*

• This study introduces a methodology using Cellular Automata (CA) models to simulate seagrass dynamics detailing conceptualization, workflow, parameterization, and execution.
• Case studies in Mediterranean and East Africa ecosystems demonstrate the versatility of CA models in capturing the impacts of climate change, invasive species, and nutrient regimes.
• Despite strengths, the CA model has limitations and uncertainties like parameterization complexity and model validations suggesting future research to enhance accuracy and applicability.

## Graphical abstract

**Figure:**
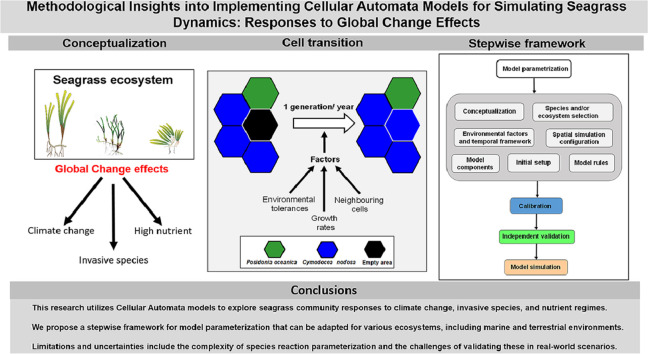

Specifications table

**Table:** 

Subject area:	Environmental Science
More specific subject area:	*Ecological modelling applied to marine plant ecosystems*
Name of your method:	Cellular automata models
Name and reference of original method:	Kubicek, A., Jopp, F., Breckling, B., Lange, C., & Reuter, H. (2015). Context-oriented model validation of individual-based models in ecology: A hierarchically structured approach to validate qualitative, compositional, and quantitative characteristics. *Ecological Complexity*, 22, 178–191.
Resource availability:	R Core Team. (2024) Oracle Corporation. (2023). Java SE Development Kit 11.

## Background

Spatial and ecological models such as species distribution models generate habitat distribution patterns based on how species respond to environmental descriptors [1]. However, these models often cannot incorporate fine-scale processes such as species interactions or heterogeneous and varying local-scale environmental and/or anthropogenic disturbances. Mechanistic models, including cellular automata (CA) can simulate ecological processes occurring at a local scale such as individual-level development and/or species interactions, providing therefore, a more comprehensive understanding of ecosystem dynamics. These models have proven particularly successful at simulating complex spatial biological and ecological processes at fine spatial scales, including plant ecosystem dynamics [[2], [3]]. Cellular automata models are discrete, stepwise, cell-oriented representations of space and time based on deterministic or probabilistic rules. These rules can describe species-environment dynamics, physiological tolerances, and complex species interactions [4].

Here we present a new ecological and spatial model using a CA approach to simulate changing seagrass distribution patterns in reaction to global climate change at a local scale, thereby utilizing two distinct case studies. The first case study focuses on Mediterranean seagrass species' responses to climate change scenarios and interactions with invasive species [5], while the second examines tropical seagrass species' responses to climate change and nutrient regimes in Zanzibar (East Africa). While our primary focus lies in delineating the steps for developing and implementing CA models for seagrass ecosystems, the methodological framework we propose can also serve as a model for implementing similar approaches for plant communities in other ecosystems or biomes.

## Method details

### Cellular automata to simulate marine plant ecosystems: A conceptual framework

Cellular automata models are dynamic representations of spatial processes, thereby using a discrete cell-based approach for the simulated spatial units. Cells have different states (discrete or continuous) and are connected to a grid with dynamics occurring through stepwise application of a rule system which changes the state of each individual cell. Spatial dynamics arise through rules considering the state of the individual focal cell to be changed and the states of cells in a defined neighbourhood.

#### Conceptualization

In a first step, it is required to clearly define the conceptual framework and specific objectives of the model, including the species or biome and the ecological processes or phenomena that are aimed to be simulated. The more clearly defined the objectives and the boundaries of the system(parts) to be represented, the easier it will be to code for the single parts and their relations. The single components of the systems and their relations can be e.g. described using a systems analysis approach and depicted in a cause-effect diagram. The framework for the model thus developed will define the general sets for the following detailed procedures.

#### Species and/or ecosystem selection

The selection of target species is crucial for accurately representing ecosystem dynamics. For instance, indicator, engineering or foundation species are potential target candidates to be included in the model because they are most relevant for the ecological interactions and form an interconnected web of relationships with other species and within the ecosystem. Additionally, the inclusion of non-native species can be relevant due to their capacity to generate shifts in native spatial population dynamics and/or biodiversity patterns.

#### Environmental factors and temporal framework

The choice of environmental variables to be considered is crucial as they shape the species' responses to the simulated scenarios. It's essential to include variables that can notably impact the selected species and ecosystems to be modelled in relation to the set objectives. Similarly, the selection of the temporal framework for simulations is vital to effectively capture temporal reactions in species and ecosystem responses and their interactions with the environment. Translated to CA-settings this relates to the duration of the simulation and even more importantly to the duration of each time step, i.e. the up-date frequency.

#### Spatial simulation configuration

The dimensions and species configuration of the model must accurately replicate a natural environment for spatial simulations to be effective. This involves considering local and regional (if applicable) environmental conditions and species distribution patterns, to adequately capture species spatial dynamics, interactions, and ecosystem processes. Furthermore, the resolution (grain, size of a single cell) of the model must be defined to best capture the representations of components.

#### Model components

The *grid* in the CA-context refers to the spatial arrangement of cells in the simulation of ecological processes, providing a structured representation of the study system, typically organized in a two-dimensional arrangement. Operating on the grid, *cells* represent the fundamental operational units with each cell posing different potential states. The geometry of cells can vary. The most common is a rectangular shape, however a hexagonal approach is preferred as it has less directional constraints. These *cell states* undergo updates from generation to generation (time-step to time-step), with each generation representing a time scale adequate for the simulated processes, through the application of defined rules. Computational constraints must be additionally considered as grids with millions of cells might take too long to up-date frequently.

#### Initial set-up

The initial set-up of a CA model involves configuring the spatial grid, defining the state variables for each cell, initializing these variables with starting values, and establishing the neighbourhood interactions. This step ensures that the model accurately represents the initial conditions and spatial structure of the ecosystem being modelled, setting the stage for subsequent simulations and analyses.

#### Rules

Defining clear and precise model rules is crucial for developing accurate simulations, as these rules determine how model species or ecosystems react to environmental factors and stressors within the defined simulated areas, and how species interact with each other. The rules must cover every situation of cell states, and states of defined neighbourhood cells. Examples of model rules include species colonization and dispersal, reproduction, biological interactions, or responses to environmental variables [[6], [7]]. The rules in CA models can be deterministic or probabilistic, depending on the nature of the ecological processes being simulated and the complexity in the simulation [[7], [8]]. *Deterministic rules:* cell state is determined by the defined rules, without any element of randomness. Therefore, changes in the state of a cell depends entirely on the current state of that cell and its neighbouring cells. *Probabilistic rules:* The cell state is determined probabilistically, allowing for the simulation of uncertainty and variability in ecological processes outcomes.

#### Model calibration in baseline scenarios

This step involves adjusting model parameters based on the available empirical data, ensuring that the model aligns with observed ecological phenomena and expert knowledge. The calibration allows researchers to validate ecological hypotheses, providing a reliable foundation for scenario testing.

#### Independent model validation

Model validation involves assessing the model's performance by comparing its outputs with empirical data from studies or temporal ecosystem responses that have been already reported. Expert knowledge and insights are critical during this stage, providing additional context and validation criteria to refine and validate the model further [9].

#### Model simulation

**Fig. 1:**
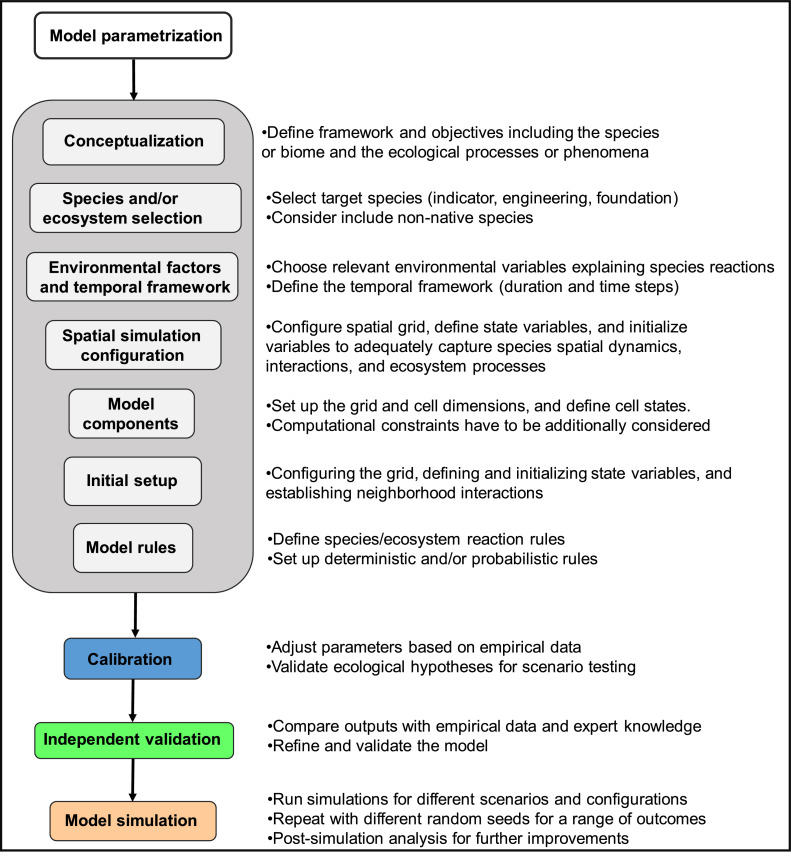
Workflow for the parametrization, calibration, independent validation, and simulation of the cellular automata (CA) model.

In this step, the simulation runs from an initial phase until a predefined endpoint or a set number of time steps, representing the final phase of the modelling process. Usually, this implies running the model for a set of different parameter configurations and environmental conditions e.g. different species configurations, scenarios with diverse environmental settings, or the inclusion of external disturbances. To get the range of potential biological relevant outcomes, the simulations for each specific setting are repeated with different random seeds. Finally, after achieving the simulation outputs the model might be analysed again for further improvement and integration of further processes (Fig. 1).

## Method validation

Here we applied the proposed CA model to two distinct case studies: one focused on Mediterranean seagrass species in response to climate change scenarios and interactions with invasive species [5], and a second focussed on tropical seagrass species' responses to nutrient regimes in Zanzibar (East Africa) [10]. These two case studies differ from each other in terms of the included species, the environmental settings, and the CA spatial settings.

## A case study in the Mediterranean seagrass ecosystems: Responses to climate change and the presence of invasive species

### Conceptualization

The primary aim of the first case study was to investigate the impact of multiple stressors, including climate change and the presence of invasive species, on the Mediterranean native seagrass community. The model was implemented in Java using the Mason framework [11].

### Mediterranean Situation: Species selection, temporal framework and spatial simulation

We chose the endemic species *Posidonia oceanica* and the native species *Cymodocea nodosa*. Other Mediterranean seagrass species with lower distribution were omitted from the model. Furthermore, the invasive species *Halophila stipulacea* was included in the model due to its increasing significance in shaping the Mediterranean seagrass community.

**Fig. 2:**
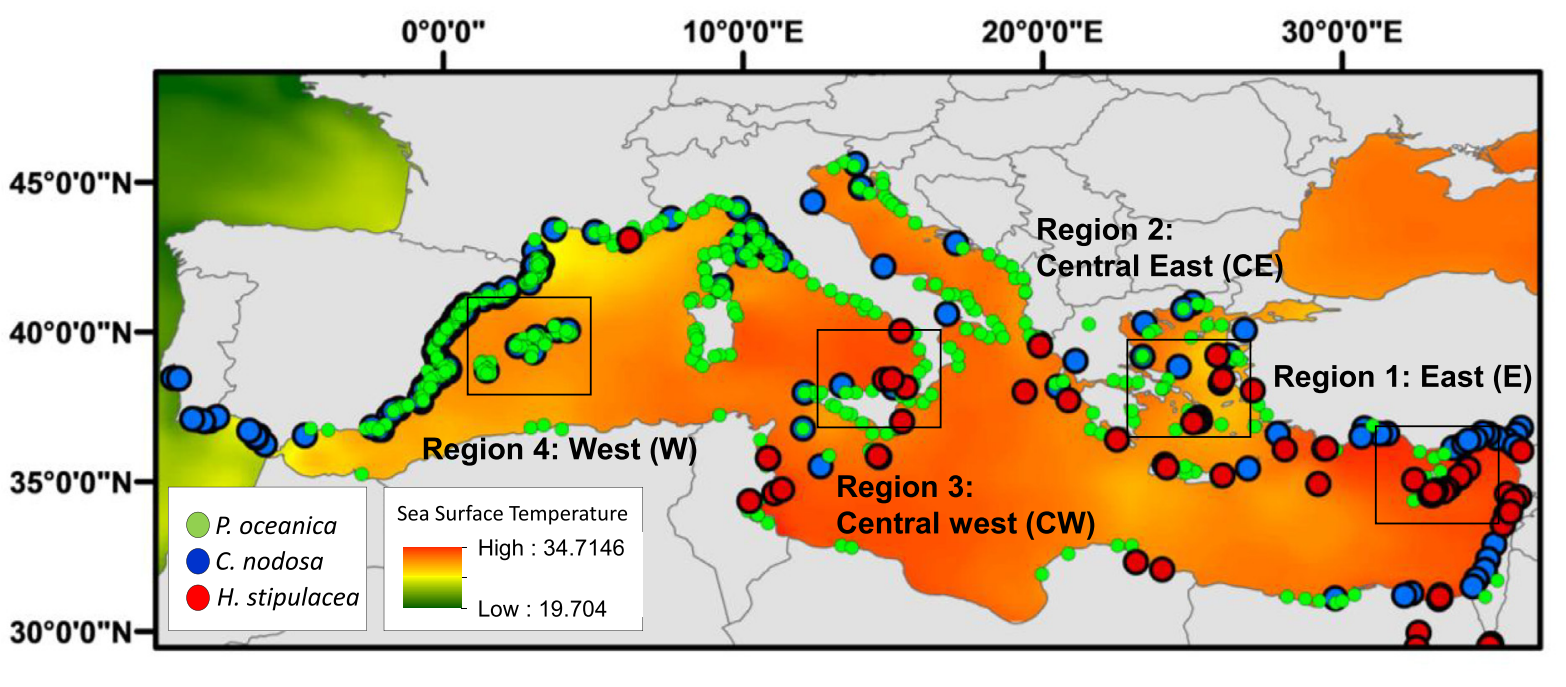
Map illustrating the existing distribution of *Posidonia oceanica, Cymodocea nodosa*, and *Halophila stipulacea* in the Mediterranean basin, along with the designated regions (highlighted by black rectangles) chosen for model implementation, encompassing the eastern (E), central eastern (CE), central western (CW), and western (W) regions. (*Adapted from*[5]).

In our study, we chose four locations from east to west in the Mediterranean Sea, representing a gradient of temperature and salinity. Specifically, the model was applied for sites with a similar latitude range (34 – 38° N): Region 1 (East [E]): 34.64042°N, 33.04051°E; region 2 (Central east [CE]): 37.00576°N, 25.28317°E; region 3 (central west [CW]): 38.28694°N, 15.59397°E; region 4 (West [W]): 38.970691°N, 1.536368°E) (Fig. 2). Simulations were conducted over an 80-year period, spanning from 2020 to 2100, with each year representing one generation.

In this study we used environmental variables of temperature and salinity available for present (2020) and future climate scenarios (2040 – 2050 and 2100) for two different greenhouse gas concentration projections including RCP 2.6 (representative concentration pathway; lowest carbon emission) and the RCP 8.5 (highest carbon emission) ([[12], [13], [14]], www.bio-oracle.org). Selecting the most divergent climate projections can better capture the range of potential variability in species reactions and distributions across different scenarios.

### Model components

For our simulations we used a hexagonal configuration of cells with each cell having an area of approximately 260cm². The cell size was determined by the annual extension rate of the three selected species and were based on the annual range of growth of the fast-growing species *C. nodosa* and *H. stipulacea*. In total, the area covered an array of 2,338m² (48.35m x 48.35m) and 90,000 cells. We used the same basic spatial layout of the grid for each simulation irrespective of the Mediterranean region. For the case study of the Mediterranean seagrass each cell can have one of five potential states defining its condition at a certain time. The states include one for each of the three species (*P. oceanica, C. nodosa* and *H. stipulacea*) and one for empty space (sand). Additionally, dead mats of *P. oceanica* are defined as the fifth state. This state is relevant as the other two species have a higher chance of colonising cells with this state.

### Mediterranean seagrass ecosystem configuration and initial setup

In this study, two primary seagrass configurations were established, both covering the same spatial extent. In the first configuration, only native species, *P. oceanica* (34%), and *C. nodosa* (41%), were incorporated into the model (*more details in section 3.4*). This configuration was applied across the four regions (east [E], central east [CE], central west [CW], and west [W]) of the Mediterranean Sea. In the second configuration, *H. stipulacea* was introduced into the model but limited to regions E, CE, and CW. In these regions, the initial seagrass landscape comprised a distribution of *P. oceanica* (34%), *C. nodosa* (25%), and *H. stipulacea* (16%). The selection of the species configuration was taken by direct observations in the Mediterranean Sea in shallow waters [[15], [16]].

### Model rules

**Fig. 3:**
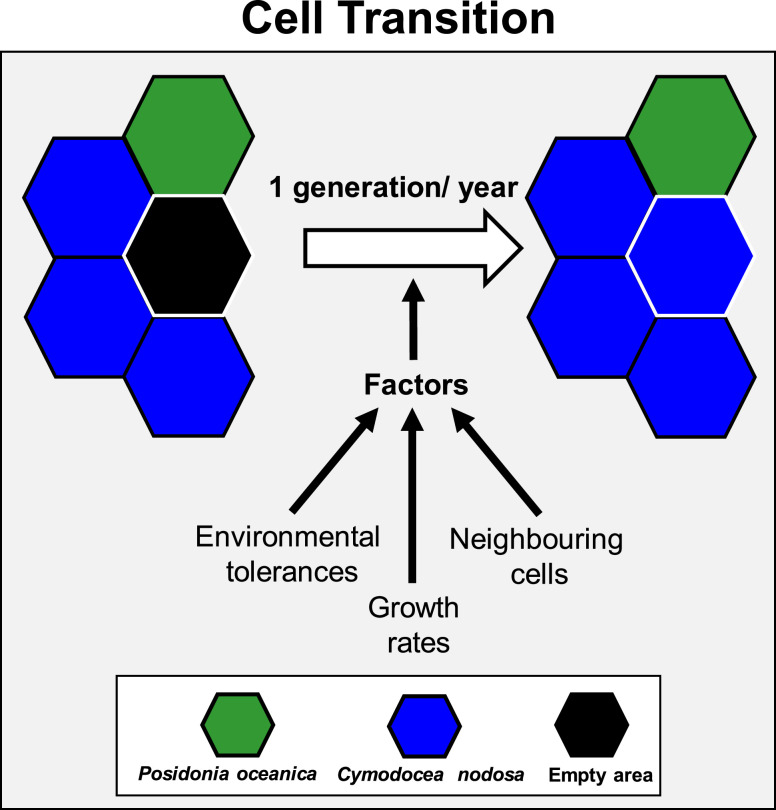
Diagram illustrating the transition of seagrass cells from one state to another within a single generation (1 year), influenced by three main factors: (i) climatic thresholds, (ii) growth rates, and (iii) the condition of adjacent cells. The third factor also considers the quantity of surrounding cells. (*Adapted from*[5]).

In the proposed model, we have developed a set of deterministic rules governing processes. Additionally, a specific degree of randomness has been incorporated into some specific processes (*details below*). In the simulations, the development of seagrass on a specific cell and the colonization of an adjacent cell depend on four factors: (i) environmental tolerances and climatic thresholds, (ii) species-specific colonization probabilities, (iii) the state and number of the surrounding neighbouring cells (Fig. 3) and (iv) mortality events.

#### Environmental tolerances and climatic thresholds

For each seagrass species, we defined three climatic domains based on minimum and maximum temperature and salinity thresholds ([13]; Assis et al., 2017). These climatic domains were categorized into: (i) optimal conditions for growth, where seagrasses (cell) thrive and can colonize an adjacent cell; (ii) suboptimal conditions for growth, where seagrasses (cell) can survive but cannot grow, and thus cannot colonize an adjacent cell; and finally, (iii) unsuitable conditions for living, where seagrasses die and the cell becomes empty, or ‘dead matte’ in the case of *P. oceanica.*

**Fig. 4:**
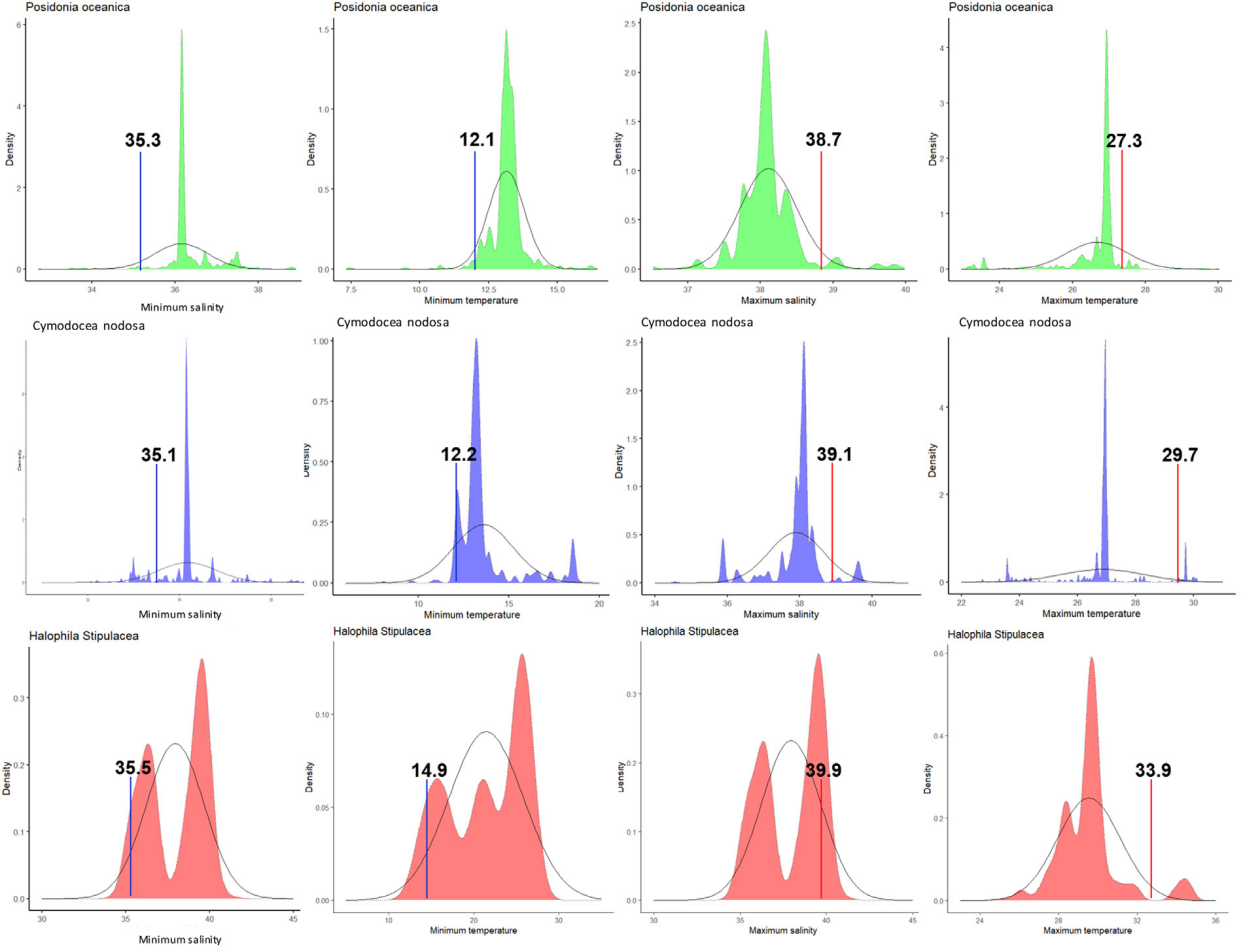
Environmental response curves were generated based on density curves depicting the maximum and minimum sea surface temperature (SST) and salinity for *Posidonia oceanica, Cymodocea nodosa,* and *Halophila stipulacea*. These curves were constructed using data from Tyberghein et al. [13] and Assis et al. [14], obtained from www.bio-oracle.org. *Posidonia oceanica* is represented by green, *Cymodocea nodosa* by blue, and *Halophila stipulacea* by red. The density distribution curve is depicted with dotted lines, while the assumed normal distribution is shown with solid lines. The 5^th^ quartile for minimum SST and salinity is indicated by blue lines, while the 95^th^ quartile for maximum SST and salinity is marked by red lines. (*Adapted from*[5])

These species-specific climatic thresholds were derived from an extensive literature survey, encompassing field observations and laboratory experiments, alongside spatial analyses. Spatial analyses involved the calculation of density curves using seagrass spatial distribution data (Fig. 4) and spatial data for both minimum and maximum sea surface temperature (SST) and salinity. These density curves were termed ‘environmental response curves.’ Subsequently, from these environmental response curves we established the three climatic domains based on quartiles. For maximum SST and salinity, optimal conditions for growth were defined as levels lower than the 95^th^ quartile; suboptimal conditions for growth were considered levels ranging between the 95^th^ and 99^th^ quartile; unsuitable conditions for living were considered levels above the 99^th^ quartile. For minimum SST and salinity, optimal conditions for growth were considered levels higher than the 5^th^ quartile; suboptimal conditions for growth were considered levels ranging between the 5^th^ and 1^st^ quartile; unsuitable conditions for living were considered levels below the 1^st^ quartile (Table 1).

**Table 1:** Average sea surface temperature (SST) and salinity thresholds for *Posidonia oceanica, Cymodocea nodosa*, and *Halophila stipulacea* were derived from both a literature review and spatial analyses utilizing environmental response curves (density curves). These averaged values, obtained from a combination of literature findings and spatial data, were directly employed in parameterizing the model.

Temperature (°)	Optimal	Suboptimal	Unsuitable
*Posidonia oceanica*	28.0	> 28-31	> 31
*Cymodocea nodosa*	30.0	> 30-33	> 34
*Halophila stipulacea*	33.0	> 33-35	> 35

Salinity (PSU)	Optimal	Suboptimal	Unsuitable

*Posidonia oceanica*	38.5	> 38,5-41	> 41
*Cymodocea nodosa*	39.0	> 39-42	> 42
*Halophila stipulacea*	43.0	nd	> 51

The final climatic threshold values were determined by averaging results from both approaches: the literature review and the environmental response curves (Table 1). These averaged values were then integrated into the model parameterization. We employed an approach similar to that used in envelope models, such as Bioclim. These models are based on an environmental envelope or range-based modeling technique, where they work by identifying the percentile range of each bioclimatic variable within which the species occurs.

Lastly, the species-specific environmental thresholds were implemented in accordance with the following scenarios: If both environmental descriptors, temperature and salinity, were within optimal conditions for growth, the environmental state for seagrasses was considered optimal. However, if one of the environmental tolerances (i.e., temperature) indicated optimal conditions for growth while the other one (i.e., salinity) was only suboptimal, the seagrass state became suboptimal. If both environmental tolerances were suboptimal, the seagrass state was unsuitable. Finally, if one environmental tolerance was optimal or suboptimal and the other one was unsuitable, the final condition turned unsuitable.

#### Species-specific colonization probabilities

In this step, we define the relationships among species and the specific type of biological interactions between them. In our model, three potential colonization types were considered: (1) a species vs. an empty cell (sand), (2) a species vs. a dead matte of *P. oceanica*, and (3) one species vs. another species (Table 2).

**Table 2:** The probabilities of colonization for each species (in %) are determined based on the various states of the neighbouring cell. These colonization probabilities are defined by considering the likelihood of a single cell colonizing a neighbouring cell.

Colonization probabilities (%)	*P. oceanica*	*C. nodosa*	*H. stipulacea*	Dead matte	Empty
*Posidonia oceanica*	-	0	0	10	5
*Cymodocea nodosa*	0	-	0	48	24
*Halophila stipulacea*	0	78	-	24	12

*Colonization of Adjacent Empty Cells:* When species interact with an empty cell (sand), we considered two options based on the described annual growth rates. In the first case, involving species with annual growth rates equal to or exceeding the cell size (0.2m), particularly *C. nodosa* and *H. stipulacea*. In the second case, applicable only to *P. oceanica*, the colonization probability was determined by dividing the annual growth rate by the cell length.

*Colonization of adjacent seagrass cells*: We assumed that the growth of *P. oceanica* remained unaffected by the presence of other seagrass species. Therefore, *C. nodosa* and *H. stipulacea* cannot colonize cells already occupied by *P. oceanica.* Similarly, *P. oceanica* cannot expand into areas where *C. nodosa* and *H. stipulacea* are present, primarily due to its low annual growth rates. The probabilities for interactions between *C. nodosa* and *H. stipulacea* were obtained from recent studies in the Mediterranean Sea based on annual coverage changes and density changes [[17], [18]].

*Colonization of adjacent dead mattes ofP. oceanica cells:* The colonization of dead mattes of *P. oceanica* was programmed for *P. oceanica, C. nodosa* and *H. stipulacea*. The probability of the modelled seagrass species to colonize a dead matte of *P. oceanica* was set to be two times higher than the probability to colonize empty cells. These assumptions were based on field studies reporting on the affinity of seagrass species to colonize this substrate [[19], [20]] and on our field observations and expertise, however no quantitative information was obtained from the literature.

#### The state and number of the surrounding neighbouring cells

Deterministic and probabilistic rules for cell transitions between different states were formulated as follows: (i) When a seagrass species occupies a cell, all surrounding empty cells are attributed an equal probability of colonization. (ii) In instances where an empty cell is surrounded by multiple cells of the same seagrass species, the colonization probability of the empty cell is multiplied by the number of adjacent seagrass cells of that species. (iii) When an empty cell is surrounded by more than one seagrass species, a weighted probabilistic approach is employed, considering the number of cells of each species and their respective colonization probabilities.

Overall, the final outcomes of cell transitions between states were generated through random simulations. These simulations took into account the defined probabilities for each species and the number of cells of each species involved. To accomplish this, we employed a Java-based algorithm designed to produce random outcomes with varying probabilities.

#### Mortality events

The processes described above can results in the mortality of a seagrass on a given cell, e.g. when the cell is colonised by another species (cell states changes to the new species) or when the environmental condition gets unsuitable (cell states changes to empty). Furthermore, the model allows to specify disturbance events, such as winter storms, anchoring or sedimentation. Each can be defined to affect a certain part of the simulation area with a certain frequency. Cell states of the target areas then turn empty with a defined probability.

### Model calibration in baseline scenarios

**Fig. 5:**
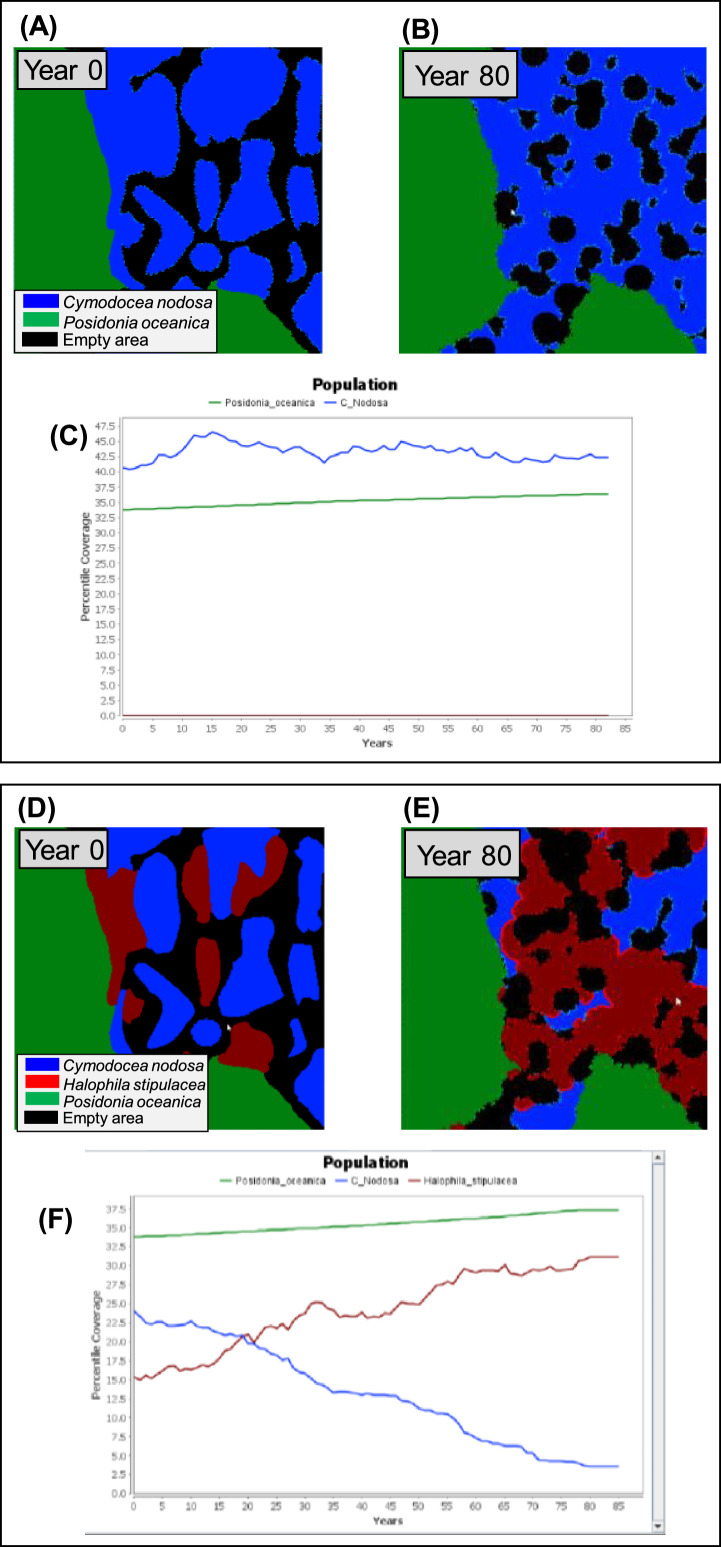
Illustration of model calibration in baseline scenarios. Panel A presents the initial configuration of the seagrass map within the Cellular Automata (CA) model, showcasing the distribution of native species, *Posidonia oceanica* (green color), and *Cymodocea nodosa* (blue color). Panel B demonstrates the final map, indicating changes in the cover (%) of each species after an 80-year simulation with constant temperature and salinity levels. Panel C represents the CA model's output, displaying the temporal trend of the cover (%) of native seagrasses over the 80-year period. Panel D exhibits the initial seagrass map configuration, showing the distribution of *P. oceanica* and *C. nodosa,* along with the invasive species *Halophila stipulacea* (red color). Panel E shows the final map, indicating changes in the cover (%) of each species following an 80-year simulation with constant temperature and salinity levels. Panel F displays the CA model's output, presenting the temporal trend of the cover (%) of both native and invasive seagrass species over the 80-year simulation period. (*Adapted from*[5]).

In this study, to establish baseline scenarios, the model underwent calibration for the present climate scenario (constant SST and salinity) with two distinct seagrass species compositions: (i) native Mediterranean seagrass species only (*P. oceanica* and *C. nodosa*), and (ii) a combination of native Mediterranean and invasive seagrass species (*H. stipulacea*) (Fig. 5).

### Independent model validation

**Fig. 6:**
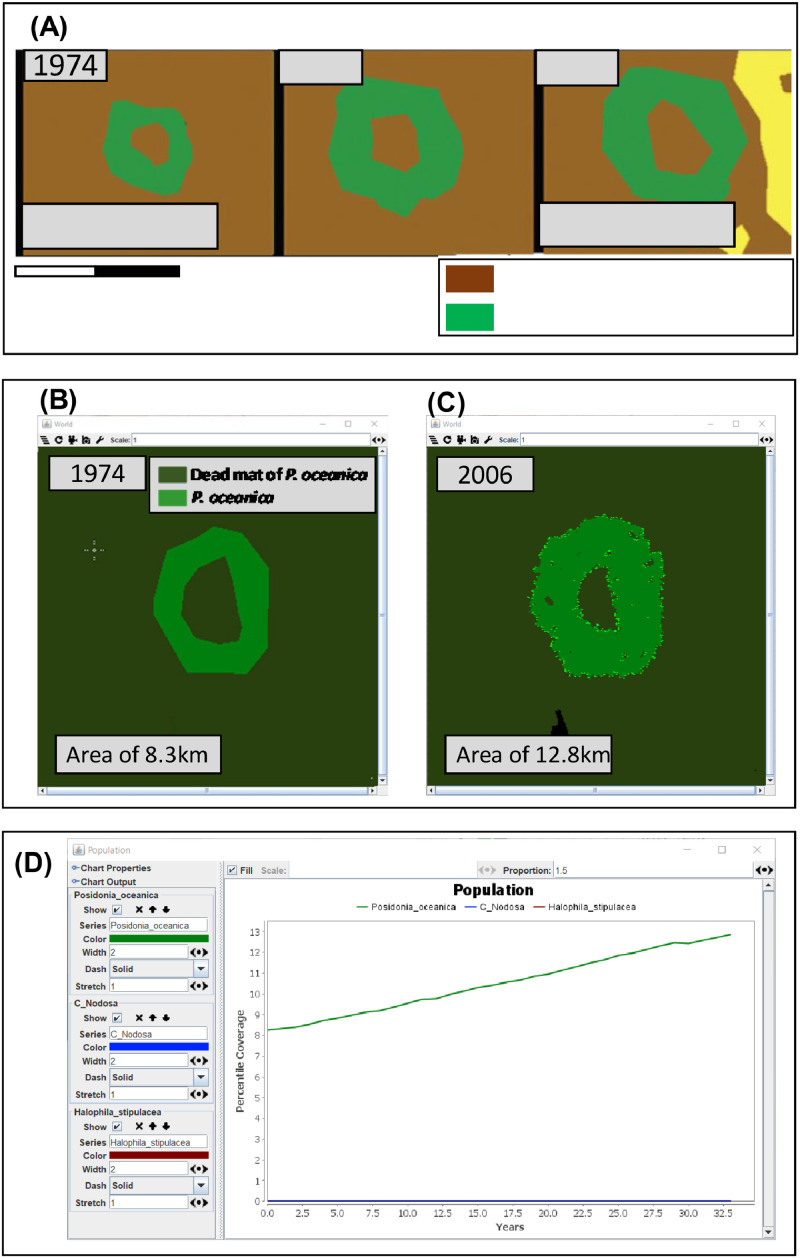
Independent model validation of *Posidonia oceanica* in real scenarios: Panel A showcases the temporal changes in the spatial distribution of *P. oceanica* in the Gulf of St Florent (Corsica, France) from 1974 to 2006, based on research by Bonacorsi et al. [21]. Panel B presents the Cellular Automata (CA) configuration of the initial seagrass map simulating the real scenario of 1974, incorporating anthropogenic disturbances. Panel C displays the final map illustrating the changes in the cover (%) of *P. oceanica*, simulating the real scenario of 2006. Finally, Panel D shows the CA output, presenting the temporal trend of the cover (%) of *P. oceanica* from 1974 to 2006. (*Adapted from*[5])

In the proposed model, the independent model validation followed the hierarchical approach outlined in Kubicek et al. [9], wherein each model level was assessed individually. Simulations underwent validation concerning specific seagrass community dynamics, encompassing scenarios with only native seagrass species [[21], [22]] (Fig. 6) and scenarios involving both native and invasive seagrass species [5].

### Model simulation

In our study, all scenario simulations were run six times with distinct random seed configurations.

**Fig. 7:**
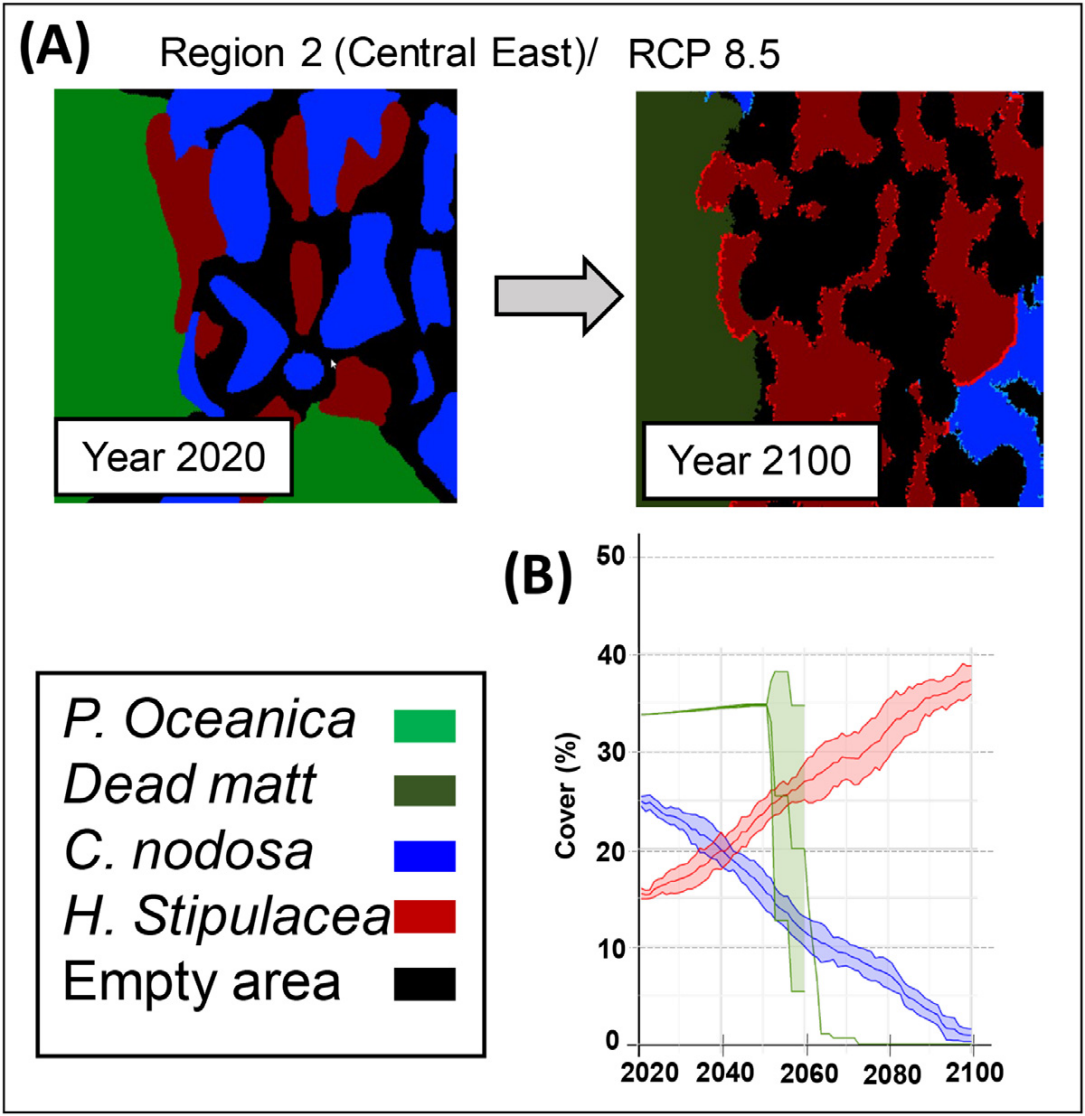
An example of model simulation in Region 2 (Central East) involving native species *Posidonia oceanica* and *Cymodocea nodosa*, along with the invasive species *Halophila stipulacea* under the RCP 8.5 climate scenario is illustrated through the following panels: Panel A presents the Cellular Automata (CA) configuration, depicting the initial seagrass map in 2020 and the final map configuration in 2100. Panel B displays a Fig. showcasing the temporal changes (±SD) in the cover (%) of the target species from 2020 to 2100. (*Adapted from*[5]).

An example of simulation runs for the Central East of the Mediterranean for the IPCC RCP 8.5 scenario (Fig. 7B). The cover of *P. oceanica* initially increases slightly before decreasing sharply after 2040 and becoming extinct between 2070-2080. *Halophila stipulacea* grows at the expense of *Cymodocea nodosa*, surpassing the coverage of the native species after 2040. By the end of the simulation (2080-2100), the native species *C. nodosa* has become almost totally displaced.

## A case study in the Zanzibar (East Africa) seagrass ecosystems: Responses to climate change and nutrient regimens

### Conceptualization

**Fig. 8:**
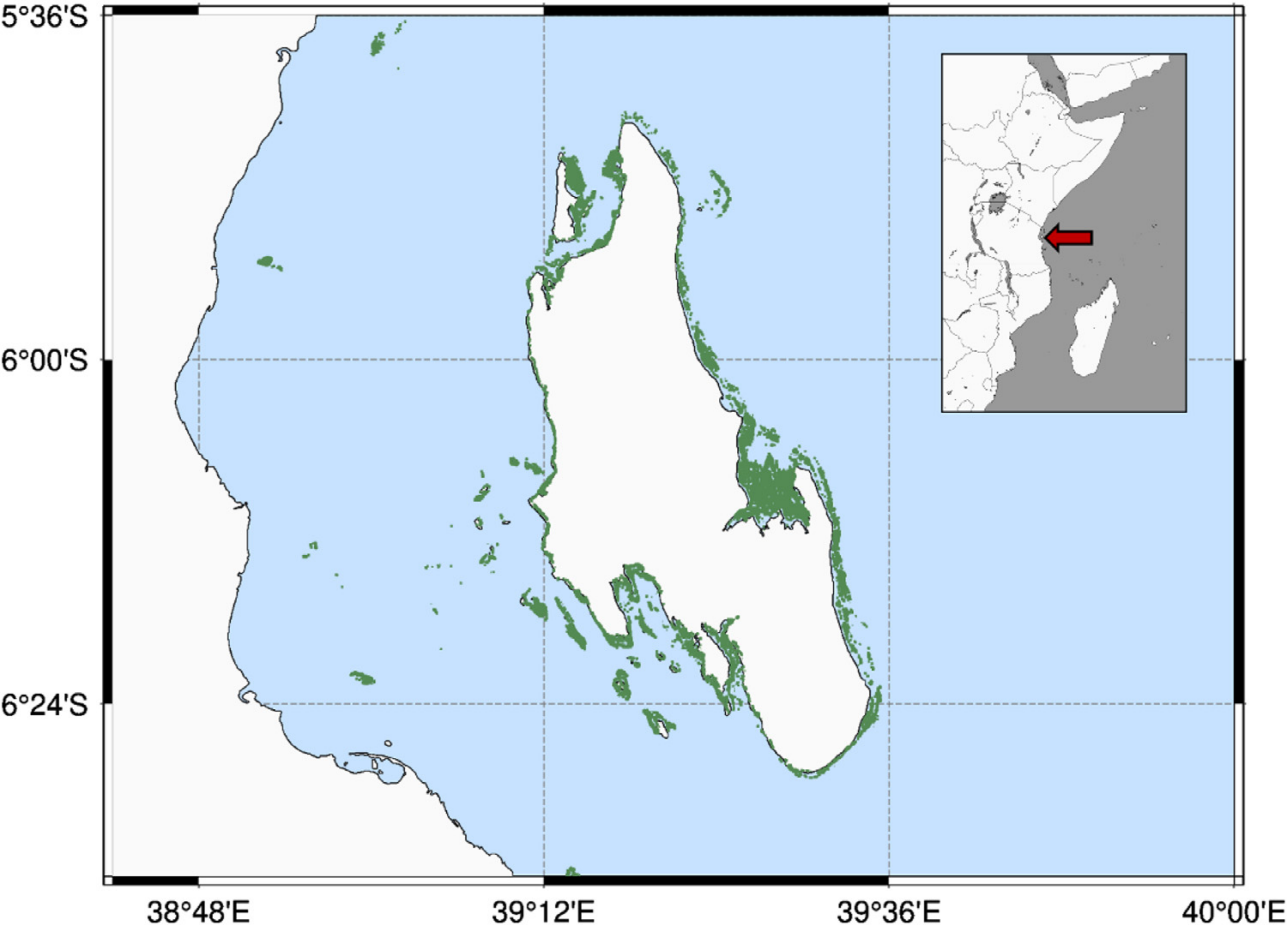
Seagrass distribution off Zanzibar. Green colour indicates the presence of seagrass [23], red arrow in locator map indicates the location of Zanzibar in front of the Tanzanian coast. (*Adapted from*[10]).

The second case study aimed to simulate seagrass development around the Island of Zanzibar, Tanzania (Fig. 8) under different temperature and nutrient concentration scenarios. Here, we emphasize the methodological aspects of model parameterization.

### Situation off Zanzibar and choice of species

The waters around Zanzibar are characterized by a particularly high diversity of seagrass, comprising 12 species. In this study, we selected three relatively abundant, co-inhabiting species with contrasting life-history strategies to be included into model simulation: *Thalassia hemprichii* (persistent), *Cymodocea serrulata* (opportunistic), and *Halodule uninervis* (colonizing). Their presence in most sites around the island, including polluted sites, make them representative of many seagrass communities in Zanzibar.

Local changes in nutrient inputs due to tourism have led to changes in seagrass cover and diversity, and rising seawater temperatures may influence this response [[12], [24]]. Two nutrient concentration scenarios (high and low) were defined and included into the model by adapting colonization probabilities and mortality to each mode. Nutrient regimes stayed constant during each simulation. Levels of nutrients were chosen based on current ambient concentrations in oligotrophic (low) and eutrophic (high) sites in Zanzibar [12].

### Model components

The model grid was defined to contain 300 × 300 hexagonal cells of 1.53 m x 1.53 m resulting in a total modelled area of 210,681 m^2^. Cell dimensions were chosen to be identical to the annual growth rate of the fastest-growing species (*C. serrulata*), facilitating the representation of growth and colonization processes. For each species, we defined two potential states based on their age: young seagrass cells (1-3 years old) and mature seagrass cells (over 2 years old).

**Fig. 9:**
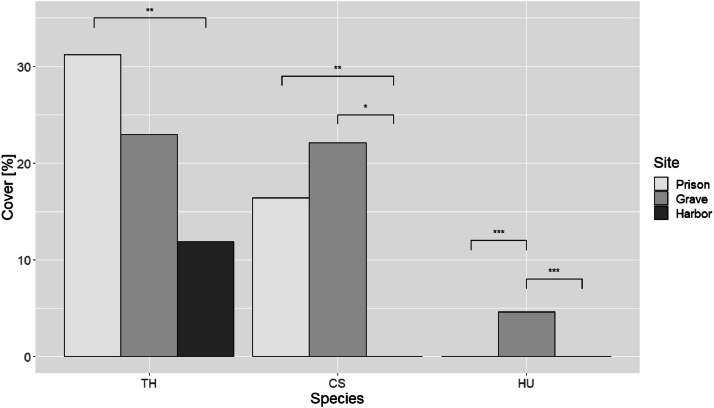
Seagrass cover of three different species at sites with different nutrient inputs (Prison = low nutrient input, Grave = intermediate nutrient input, Harbor = high nutrient input). Species are abbreviated as follows: TH = *Thalassia hemprichii*, CS = *Cymodocea serrulata*, HU = *Halodule uninervis*. Sites are abbreviated as Prison = Prison Island, Grave = Grave Island, and Harbor = Stone Town Harbor. Pairwise comparisons per species were generated using Dunn's post-hoc test with Bonferroni correction. (*Adapted from*[10]).

Each cell could be exclusively occupied by one of the three species, resulting in a total of seven possible cell states: young seagrass cell (1-3 years old), mature seagrass cell (over 2 years old), and one for empty space (sand) (7). An empty cell (sand) refers to a cell within the CA grid that has the potential to be colonized by any species of seagrass (Fig. 9).

**Fig. 10:**
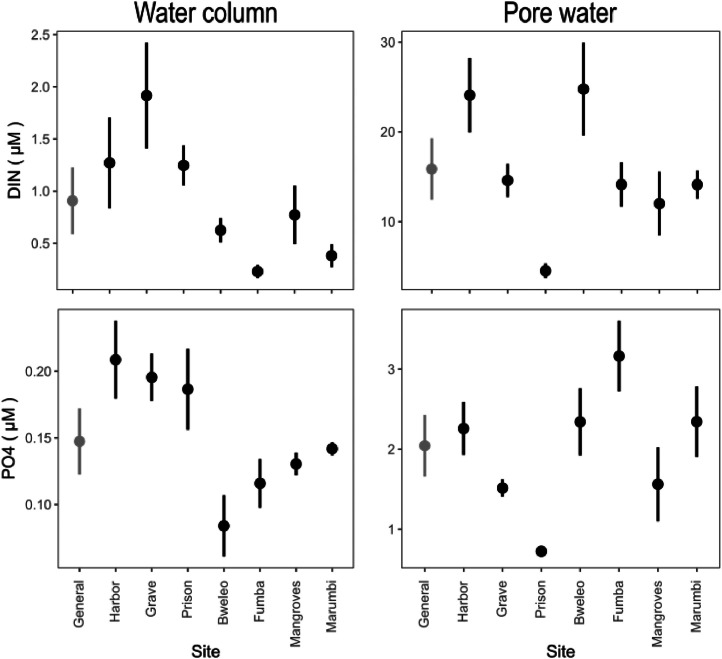
Nutrient concentrations in the water column and the pore water in sites around Unguja Island (Zanzibar Archipelago).

In this study, we selected three sites characterized by different nutrient regimes, forming a nutrient enrichment gradient from the Harbor site (located at the mouth of a sewage discharge) to Grave and Prison Islands, which are 3.5 and 5.5 km offshore, respectively. Dissolved inorganic nitrogen (DIN) and phosphate (PO4) show a significant reduction in their concentrations [(DIN: F (2, 47) = 11.367, p<0.001; PO4: F (2, 47) =11.837, p<0.001)] in the pore water from the Harbor site to Grave (DIN: t=2.279, p=0.027; PO4: t=2.321, p=0.024) and Prison (DIN: t=4.767, p<0.001; PO4: t=2.470, p=0.017) Islands (Fig. 3, right column). This pattern is not reflected in the water column values [(DIN: F (2, 27) = 0.898, p=0.418; PO4: F (2, 27) = 0.178, p=0.837)], which are more variable (Fig. 10, left column). This variability is generally due to the rapid uptake of nutrients by phytoplankton, macroalgae, and seagrass in the water column.

### Initial setup

**Fig. 11:**
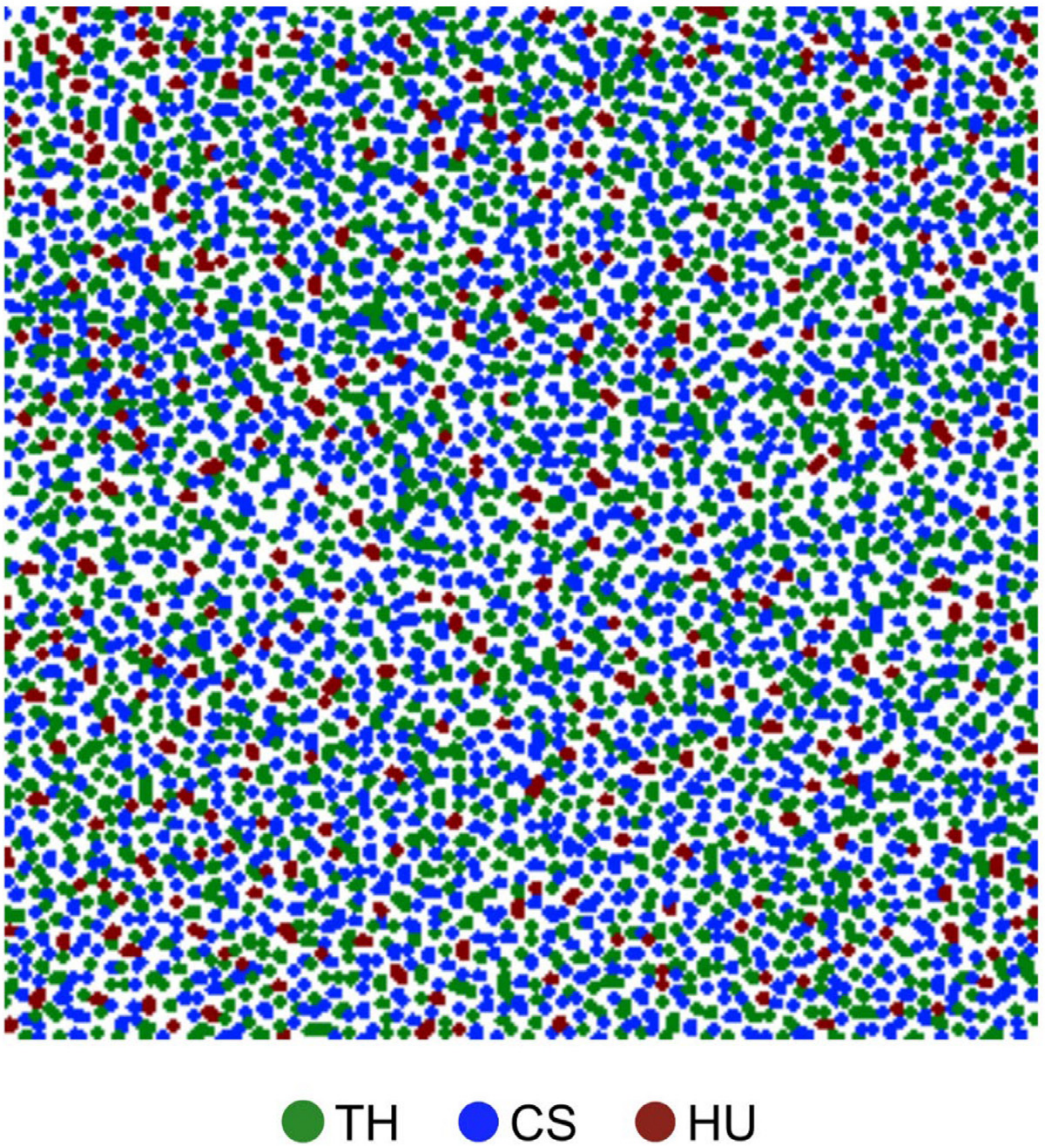
Map illustrating the initial distribution of seagrass cover used for model simulations in the Zanzibar case study. Green = T. hemprichii, Blue = C. serrulata, Red = H. uninervis (Adapted from [10]).

Initial seagrass coverage was defined based on data collected off Chapwani (grave) Island, Zanzibar, a site with rather low nutrient input where all three species co-occur [[10], [12]]. As indicated by the field data, total seagrass cover at the beginning of model simulation was defined to be 58%. The coverage for each species was determined by scaling up their individual proportions, as indicated by the field data, to achieve the total percentage coverage when combined. This resulted in final coverages of 26% (*T. hemprichii*), 26% (*C. serrulata*), and 6% (*H. uninervis*). An image file mapping the initial seagrass coverage was created based on the upscaled proportions, with seagrasses defined to occur in heterogeneous aggregations of 2–10 meters lengths (Fig. 11).

### Model Rules

As described for the simulation of Mediterranean seagrass ecosystems, simulation of seagrass development off Zanzibar was primarily governed by a set of deterministic rules referring to (i) environmental tolerances and climatic thresholds, (ii) species-specific colonization probabilities, (iii) the state and number of the surrounding neighbouring cells (iv) mortality events.

We first determined parameters assuming a low nutrient concentration scenario. In a second step we changed both colonization probabilities and mortality to mimic conditions under high nutrient input.

#### Seagrass mortality under different nutrient regimes

##### Mortality under low nutrient regime

Mortality events were simulated by the CA by turning cells covered with seagrass into empty cells. We used a ranking provided by [25], on species-specific resistance to mechanical disturbances as a proxy to define different mortality rates, as available quantitative data was not sufficient. We estimated mortality rates under low nutrient regime experimentally for each species during model calibration (see Section 4.4. for details) with seagrass mortality being the lowest in *T. hemprichii* (8 – 9% per year), intermediate in *H. uninervis* (9 – 12% per year) and highest in *C. serrulata* (20 – 22% per year).

##### Mortality under high nutrient regime

To simulate seagrass development under high-nutrient conditions, we utilized field measurements from Stonetown Harbor, Zanzibar, a site with high nutrient input, as a reference (Fig. 10). These data showed decreased coverage of all species compared to sites with low nutrient input. To mimic the conditions at Stonetown Harbor we uniformly increased the mortality of all species until the coverage of *T. hemprichii* declined to 12%. Final mortality rate for each species under high-nutrient conditions was raised by approximately one third compared to that defined for low-nutrient conditions.

#### Species-specific colonization probabilities under different nutrient regimes

We considered two colonization types: (1) one species vs. an empty cell and (2) one species vs. another species. Colonization probabilities of one species to an empty cell were derived using rhizome elongation rates found in the literature [26], while colonization probabilities to cells occupied by other species were approximated experimentally during model calibration.

##### Colonization of adjacent empty cells under low nutrient regime

The colonization probabilities for empty cells, assuming a low nutrient regime, were determined based on rhizome elongation rates as reported by Marbà and Duarte [26]. This calculation considered two main factors: (i) cells within the grid were standardized to have a length of 1.53 m, matching the annual horizontal growth of the fastest-growing species, *C. serrulata*; (ii) each cell on the grid was surrounded by six neighboring cells. Consequently, the colonization probability for *C. serrulata* was calculated as 100% / 6 = 17%. The colonization probabilities for other species were adjusted proportionally (Table 3).

**Table 3:** Colonization probabilities for each species (in %) in relation to the states of the neighbouring cells. Brackets indicate the increased colonization probability of *T. hemprichii* in the high nutrient scenario.

Colonization probabilities (%)	*T. hemprichii*	*C. serrulata*	*H. uninervis*	Empty
*Thalassia hemprichii*	-	4	2	6 (15.6)
*Cymodocea serrulata*	4	-	5	17
*Halodule uninervis*	0	0	-	11

##### Colonization of adjacent empty cells under high nutrient regime

To gain information on colonization under high nutrient regime, we conducted fieldwork at sites with varying nutrient inputs off Zanzibar, measuring rhizome elongation rates [10]. The results indicated a 2.6-fold increase in horizontal growth for *T. hemprichii*, despite a small sample size for all species. Based on these findings, we increased the colonization probability of *T. hemprichii* to empty cells by 2.6 times in simulations of high nutrient regimes.

##### Colonization of adjacent seagrass cells

Colonization probabilities from one cell to an adjacent cell occupied by another species were defined experimentally during model calibration after setting all other parameters, as available quantitative information on species interactions was not sufficient. Colonization probabilities were selected to ensure stable conditions, while approximately aligning with qualitative data on the competitive strength of the various species. Colonization of adjacent seagrass cells did not change under a high nutrient regime.

### Model validation

Due to little information on seagrass species development in the Western Indian Ocean, the model could not be validated in this stage using field evidences.

### Model calibration in baseline scenarios

**Fig. 12:**
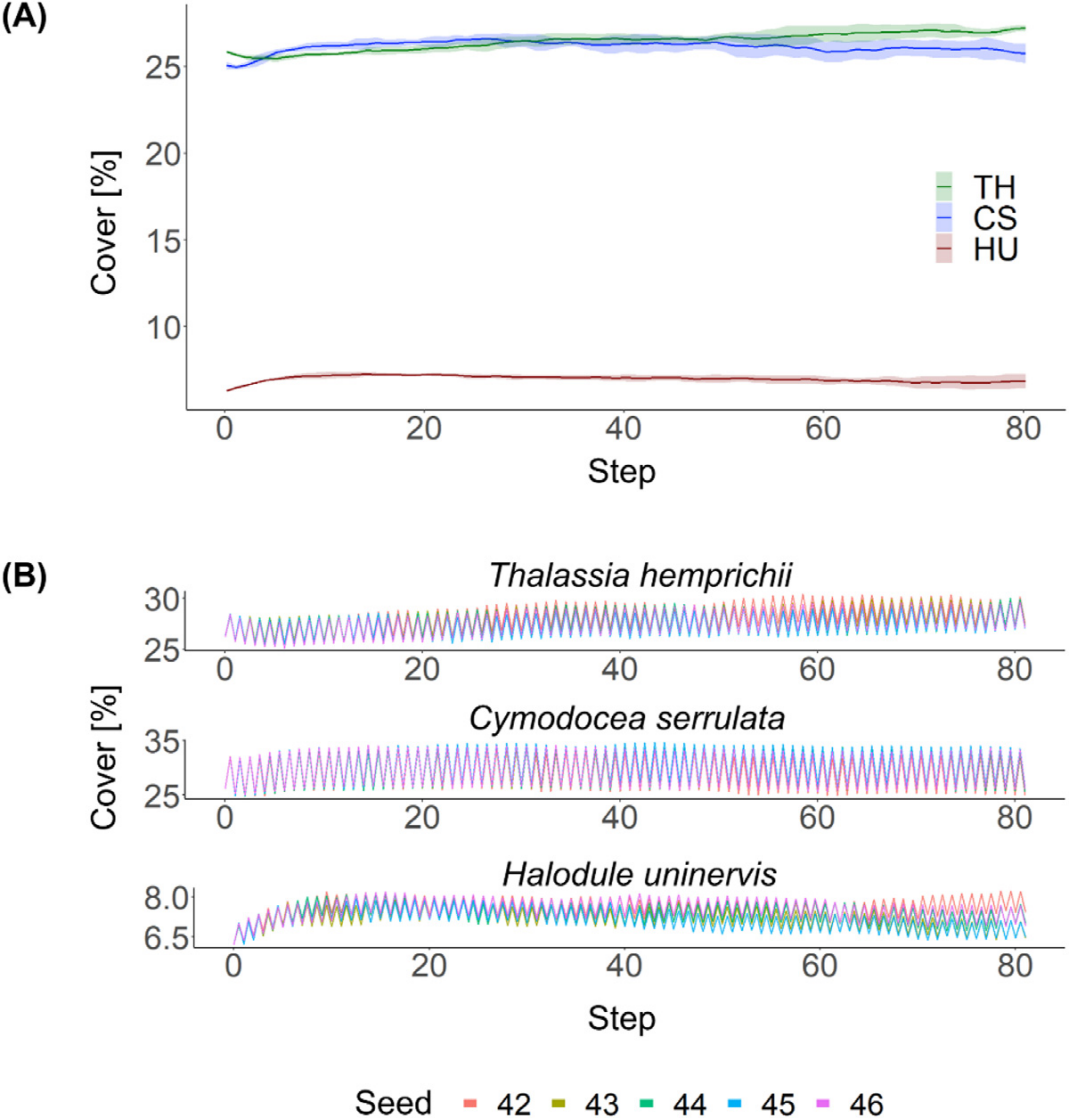
Panel A shows the seagrass cover as a function of time after model calibration. The yearly output of for all five simulation runs per species was averaged. Panel B displays the seasonal output of simulation runs for model calibration using five different seeds (42 – 46). Each step represents one year. Growth of seagrass took place during first half of the year whereas death of seagrass occurred mostly in the second half. TH = *T. hemprichii*, CS = *C. serrulata*, HU = *H. uninervis* (*Adapted from*[10]).

The aim of the calibration was to maintain the cover of each species relatively constant during the entire simulation period. Parameters for which qualitative data were available (initial conditions, colonization probabilities to empty cells) were set first. Then, parameters for which proportional data were available (mortality of each seagrass species) were defined experimentally, but in approximation to information found in the literature. Subsequently, parameters for which only qualitative information was available (colonization probabilities to occupied cells) were estimated experimentally. The model was run five times with varying seeds at constant temperature conditions for 80 years (Fig. 12A). The model was fine-tuned by slightly adjusting two parameters (frequency of disturbances and colonization probability to occupied cells) until stable conditions were achieved and variability between seeds was minimized (Fig. 12B).

### Model simulation

**Fig. 13:**
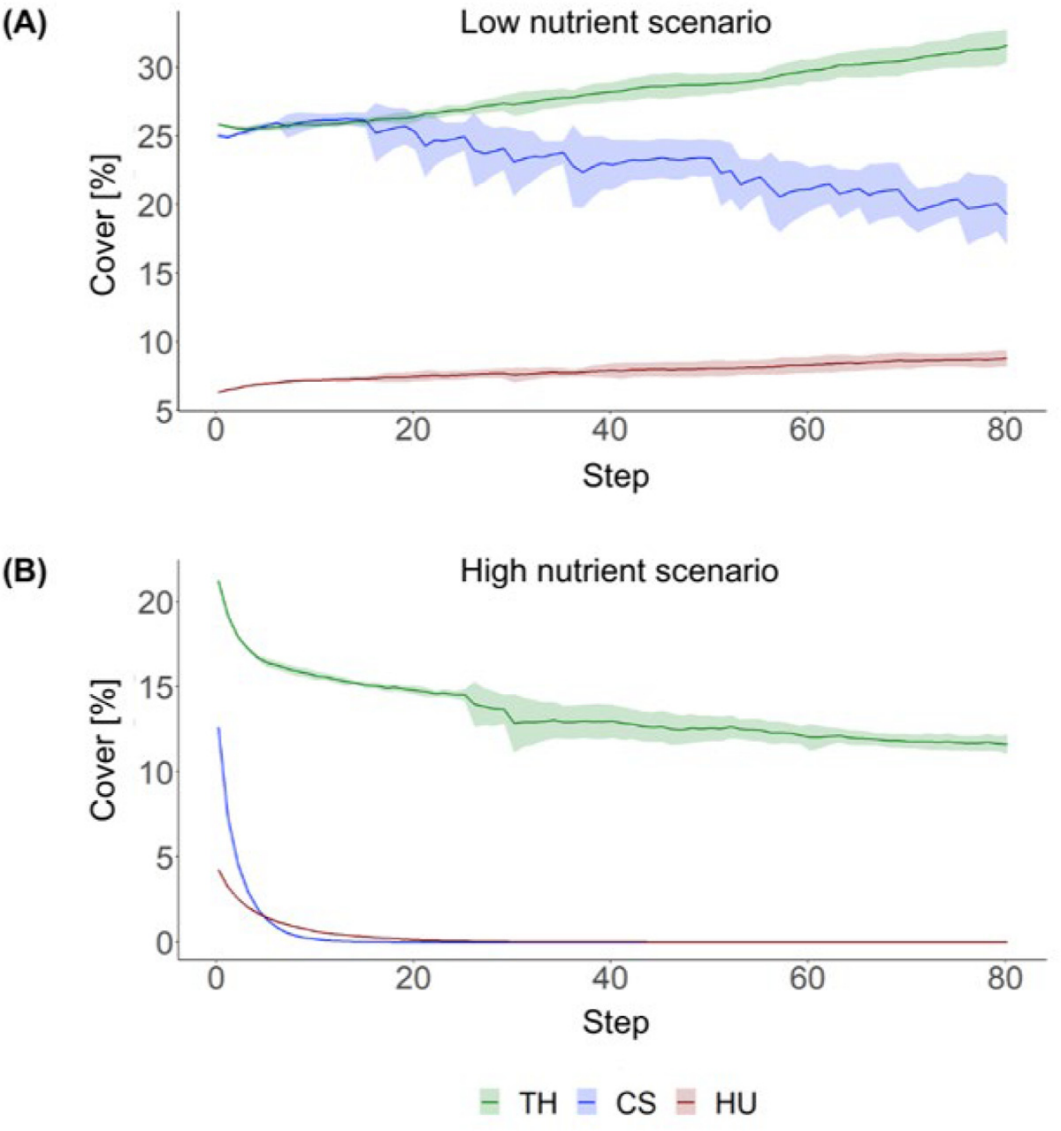
Development of cover of three seagrass species under (A) low and (B) high nutrient regime. TH = *T. hemprichii*, CS = *C. serrulata*, HU = *H. uninervis*. Simulation was conducted five times and results were averaged. (*Adapted from*[10]).

To simulate seagrass development off Zanzibar, the CA was run under two different nutrient scenarios, assuming an RCP 2.6 temperature scenario. Each nutrient scenario was simulated five times with varying seeds to compare the temporal development of seagrass coverage. In the low nutrient scenario, species cover remained relatively stable, with *T. hemprichii* and *H. uninervis* showing slight upward trends, and *C. serrulata* showing a downward trend. Minor changes occurred due to heatwaves and species interactions (Fig. 13A). In contrast, the high nutrient scenario led to an immediate decrease or extinction of all seagrass species, highlighting the severe impact of high nutrients on seagrass development. *T. hemprichii* was the most resistant to high nutrients but still experienced a decline in cover of around 50 % (Fig. 13B). *C. serrulata* and *H. uninervis* were highly vulnerable to increased nutrients, with both species going extinct within the first 20 steps of the simulation.

In conclusion, the results of our models demonstrate the robustness of CA models in simulating real-case scenarios and projecting future trends and nutrient stress conditions. Moreover, our stepwise approach to model parameterization offers a structured framework that can be adapted and applied to seagrass or other marine or terrestrial ecosystems from different geographical regions, species compositions, and environmental settings.

## Limitations and uncertainties

It is important to acknowledge the limitations and uncertainties inherent in our model. These include the complexity of parameterization such as assumptions regarding environmental thresholds or species explanatory variables, the acquisition of growth rates of target species and their interactions, and the lack of comprehensive data on colonization capacity. Additionally, we encountered challenges in finding real-world cases in the literature for model validation. Overall, we observed that there is often a significant lack of field or experimental data on species' responses to environmental variables.To address this, we propose implementing spatial and environmental analyses to establish the environmental thresholds needed for accurate model implementation. For example, during the parameterization of the Zanzibar model, significant data gaps were identified, particularly in the areas of (1) colonization probabilities, (2) species interactions, and (3) mortality rates under varying nutrient conditions. Due to the absence of sufficient data, these parameters were primarily determined experimentally, which, in turn, increased the uncertainty of the model's results. Another limitation is the lack of studies available to validate our simulations. For instance, in the study by Meister [10], the outcomes derived from Cellular Automata (CA) models were compared with the results of a species distribution model conducted in the same area. This comparison provided a form of validation against other models. Furthermore, there are computational constraints associated with simulating spatial grids. To accurately capture fine-scale processes, as proposed in our models, it is necessary to work with small grid cells. However, this requirement makes it impractical to conduct simulations over large areas, such as the entire Mediterranean Sea. As an alternative, we simulated our models in four different Mediterranean regions, each representing a broad range of environmental conditions. Another source of uncertainty is related to the choice of temperature, salinity, and nutrient availability as the main environmental descriptors. While crucial, these factors might not capture other important influences, such as hydrodynamic forces or pH levels, which can also affect seagrass species' reactions and distributions. Lastly, RCP 2.6 and RCP 8.5 represent vastly different futures, assuming either significant mitigation efforts or continued high emissions. However, real-world outcomes might not align perfectly with these assumptions due to unexpected socioeconomic, political, or technological changes.

Addressing these limitations in future research endeavors is crucial for enhancing the accuracy and flexibility of model parameterization and generating more reliable simulations. By doing so, we can deepen our understanding of ecosystem dynamics and contribute to more effective conservation and management efforts worldwide.

## CRediT authorship contribution statement

**Pedro Beca-Carretero:** Conceptualization, Methodology, Validation, Data curation, Supervision, Writing – original draft, Funding acquisition. **Marlene Meister:** Methodology, Validation, Data curation, Writing – original draft. **Mirta Teichberg:** Funding acquisition, Supervision, Writing – review & editing. **Agustin Moreira-Saporiti:** Writing – review & editing, Data curation. **Fabian Schneekloth:** Software. **Hauke Reuter:** Conceptualization, Methodology, Supervision, Funding acquisition, Writing – review & editing.

## Declaration of competing interest

The authors declare that they have no known competing financial interests or personal relationships that could have appeared to influence the work reported in this paper.

## Data Availability

Data will be made available on request.

## References

[1] Elith J., Leathwick J.R. (2009). Species distribution models: Ecological explanation and prediction across space and time. Annu Rev. Ecol. Evol. Syst..

[2] Breckling B., Peer G., Matsinos Y.G. (2011). Modelling Complex Ecological Dynamics: An Introduction into Ecological Modelling for Students, Teachers & Scientists.

[3] Mayol E., Boada J., Pérez M., Sanmartí N., Minguito-Frutos M., Arthur R., Alcoverro T., Alonso D., Romero J. (2022). Understanding the depth limit of the seagrass *Cymodocea nodosa* as critical transition: Field and modeling evidence. Mar. Environ. Res..

[4] Schonert T., Milbradt P. (2005). Computing in Civil Engineering.

[5] Beca-Carretero P., Winters G., Teichberg M., Procaccini G., Schneekloth F., Zambrano R.H., Reuter H. (2024). Climate change and the presence of invasive species will threaten the persistence of the Mediterranean seagrass community. Sci. Total Environ..

[6] Colasanti R.L., Grime J.P. (1993). Resource dynamics and vegetation processes: A deterministic model using two-dimensional cellular automata. Funct. Ecol..

[7] Li H., Reynolds J.F. (2023). In Scale in Remote Sensing and GIS.

[8] Decocq G., Regnault P., Lenoir J., Paccaut F., Di Menza L., Delvoye G., Goubet O. (2023). Modelling plant community dynamics in changing forest ecosystems: A review. Botany Lett..

[9] Kubicek A., Jopp F., Breckling B., Lange C., Reuter H. (2015). Context-oriented model validation of individual-based models in ecology: A hierarchically structured approach to validate qualitative, compositional, and quantitative characteristics. Ecol. Complex..

[10] Meister M. (2021).

[11] Luke S., Cioffi-Revilla C., Panait L., Sullivan K., Balan G. (2005). Mason: A multi-agent simulation environment. Simulation..

[12] IPCC, Masson-Delmotte V., Zhai P., Pirani A., Connors S.L., Péan C., Chen Y., Goldfarb L., Gomis M.I., Matthews J.B.R., Berger S., Huang M., Yelekçi O., Yu R., Zhou B., Lonnoy E., Maycock T.K., Waterfield T., Leitzell K., Caud N. (2021). Contribution of Working Group I to the Sixth Assessment Report of the Intergovernmental Panel on Climate Change.

[13] Tyberghein L., Verbruggen H., Pauly K., Troupin C., Mineur F., De Clerck O. (2012). Bio-ORACLE: A global environmental dataset for marine species distribution modelling. Glob. Ecol. Biogeogr..

[14] Assis J., Tyberghein L., Bosch S., Verbruggen H., Serrao E.A., De Clerck O. (2018). Bio-ORACLE v2.0: Extending marine data layers for bioclimatic modelling. Glob. Ecol. Biogeogr..

[15] Gambi M.C., Barbieri F., Bianchi C.N. (2009). New record of the alien seagrass *Halophila stipulacea* (Hydrocharitaceae) in the western Mediterranean: A further clue to changing Mediterranean Sea biogeography. Marine Biodiversity Records.

[16] Nguyen H.M., Savva I., Kleitou P., Kletou D., Lima F.P., Sapir Y., Winters G. (2020). Seasonal dynamics of native and invasive *Halophila stipulacea* populations: A case study from the northern Gulf of Aqaba and the eastern Mediterranean Sea. Aquat. Bot..

[17] Chiquillo K.L., Barber P.H., Vasquez M.I., Cruz-Rivera E., Willette D.A., Winters G., Fong P. (2023). An invasive seagrass drives its own success in two invaded seas by both negatively affecting native seagrasses and benefiting from those costs. Oikos..

[18] Sghaier Y.R., Zakhama-Sraieb R., Charfi-Cheikhrouha F. (2014). Proceedings of the 5th Mediterranean Symposium on Marine Vegetation.

[19] Almela E.D., Marbà N., Alvarez E., Santiago R., Martínez R., Duarte C.M. (2008). Patch dynamics of the Mediterranean seagrass *Posidonia oceanica*: Implications for recolonisation process. Aquat. Bot..

[20] Wesselmann M., Chefaoui R.M., Marbà N., Serrao E.A., Duarte C.M. (2021). Warming threatens to propel the expansion of the exotic seagrass Halophila stipulacea. Front. Mar. Sci..

[21] Bonacorsi M., Pergent-Martini C., Breand N., Pergent G. (2013). Is *Posidonia oceanica* regression a general feature in the Mediterranean Sea?. Mediterr. Mar. Sci..

[22] Burgos E., Montefalcone M., Ferrari M., Paoli C., Vassallo P., Morri C., Bianchi C.N. (2017). Ecosystem functions and economic wealth: Trajectories of change in seagrass meadows. J. Clean. Prod..

[23] Allen Coral Atlas. (2022). Imagery, maps, and monitoring of the world's tropical coral reefs.

[24] Purvis D., Jiddawi N. (2023). Seagrass cover reduction in Zanzibar from 2006 to 2019. Western Ind. Ocean J. Marine Sci..

[25] Birch W., Birch M. (1984). Succession and pattern of tropical intertidal seagrasses in Cockle Bay, Queensland, Australia: A decade of observations. Aquat. Bot..

[26] Marbà N., Duarte C.M. (1998). Rhizome elongation and seagrass clonal growth. Mar. Ecol. Prog. Ser..

